# Dublin Hospital Reports

**Published:** 1827-10

**Authors:** 


### MEDICO-CHIRURGICAL REVIEW.

(FOURTH NUMBER OF 1827.)

"Nec tibi quid liceat sed quid fecisse decebit
"Occurrat, mentemque domat respectus honesti."

Vol. VII.] OCTOBER 1, 1827. [No. 14.

[NEW SERIES.]

### I.

*The Dublin Hospital Reports, and Communications in Medicine and Surgery.* Volume the fourth. Dublin, 1827.

In our last Number, we gave an analysis of several articles in this volume; and we now renew our labours, in order to diffuse through the profession at large the results of hospital practice in the Sister Isle.

### Art. I.

*Report of the Amputation of Portions of the Lower Jaw, performed at Steevens's Hospital.* By James W. Cusack, A.B. M.D. &c. &c.

This formidable operation has lately become rather familiar to the surgical world in France, in America, and in Great Britain. Those diseases requiring amputation of the inferior maxilla are either cancerous affections commencing in the soft parts, and contaminating the bone by contiguity—or morbid growths, originating in the medullary structure, and endangering life by their effects on the constitution, or interference with the functions of neighbouring organs. The operation in question, even at the articulation, is not so formidable a one as might be supposed—nor is it attended with so much inconvenience or deformity as might naturally be expected. But we must proceed at once to give some account of the cases, seven in number, which are contained in this paper.

*Case* 1. A woman, aged 46, of healthy constitution, was admitted into the Meath hospital, on the 21st of June, 1824, stating that, six years previously, after the extraction of a molar tooth, a small tumour rose from the vacant alveolar space, being firm and elastic to the touch, and nearly insensible to pressure. When it had become somewhat larger, an apothecary pushed a lancet into its centre, which was followed by profuse hæmorrhage. Other practitioners made other incisions, and all were followed by much bleeding. In the course of a year, the swelling occupied a large portion of the bone, impeding the motions of the tongue, and preventing mastication. Still the patient was free from pain. A deep incision now was followed by a rapid extension of the disease, which impeded respiration and deglutition—was attended with profuse ptyalism and occasional hæmorrhages, and in this state she entered the hospital.

"When she presented herself at the hospital, I found almost the entire of the left side of the lower jaw involved in the disease,—the tumour projected outwards, and caused much deformity, the jaws being separated by a portion which protruded between the teeth, and prevented the closure of the lips. On inspecting the cavity of the mouth, the tumour appeared to consist of three branches, involving the bone in their centre; the first, or outer, formed the prominence which was visible externally; the second, ascending between the upper jaw and cheek, and distorting the countenance, reached as high as the margin of the orbit; the third, filling up the sublingual cavity, elevated the tongue, and pushed it to the opposite side: this portion likewise extended so far backwards as to press on the anterior arch of the palate. The teeth were observed on the surface of the tumor, sunk into its substance, and perfectly moveable. The entire portion of the jaw, included between its angle on the left side, and the last incisor tooth on the right, was more or less affected.

"The situation and extent of the disease will explain the nature of her sufferings. She complained of difficulty of respiration and deglutition, articulated indistinctly, and was unable to take nourishment, except in a liquid state. A continued discharge of saliva, mixed with bloody sanies, streamed over that portion of the tumor which protruded from her lips. Her general health, however, might be considered good;—she had not yet suffered much constitutional disturbance, and preserved a tolerable appetite. Under these circumstances, the rapid increase of the disease threatening soon to put a period to her existence, it was decided, in consultation, to attempt the removal of the diseased portion of bone, as the only measure that afforded a prospect of saving the patient's life.

"On Friday, July 7th, I proceeded to the operation, in presence of the surgeons of the hospital, Messrs. Crampton, Peile, Colles, and Wilmot.—The patient was seated on a chair, her head supported, and inclined to the left side: this position being deemed preferable to the recumbent posture, as best calculated to favor the escape of the blood, and prevent its accumulation in the fauces.

"Standing before the patient, I commenced by making an incision from the commissure of the lips, on the right side, which, passing obliquely downwards, and dividing the parts completely through, terminated about half an inch below the base of the jaw; the bone being thus laid bare, was divided by a small handsaw through the alveolar process of the right canine tooth, which had been previously extracted. The next incision extended from the lobe of the ear, in the direction of the ramus, to the angle of the jaw; and both were connected by a third, carried parallel to the base. Dissecting up the flap thus formed, I divided the masseter muscle, which was expanded over the arterior surface of the tumor; and denuded the bone midway between its angle and condyle. A needle, to which the chain saw had been connected, was passed behind the ramus, the point being kept close to the bone; the saw moved with so much ease and freedom, that the patient did not appear sensible of the division of the nerve. I then pressed the tumor downwards, to put the soft parts (attached to the bone internally) on the stretch; and concluded the operation, by cutting across the muscles connected with the base of the jaw, exactly at their point of insertion.

"The hemorrhage was inconsiderable; the dental, and some branches of the facial artery, were secured by ligatures; dossils of lint were placed in the cavity, to give support to the cheek; and the divided parts brought into apposition, and retained by points of interrupted suture. A light dressing was applied, and the entire supported by a bandage and compress.

"The operation was tedious, yet the patient appeared so little exhausted by its duration, or the loss of blood, that she was able immediately after to walk to her bed. She swallowed drink in small quantities, which was conveyed to the base of the tongue by means of a gum elastic tube, attached to the spout of a teapot." 8.

Her recovery was rapid—complete union of the divided soft parts followed—and, on the 12th day from the operation, she was able to walk about the wards. Scarcely any deformity was ultimately observable. In six weeks she returned to the country in good health.

*Case* 2. A strong healthy boy, aged 12, was admitted Sept. 4, 1824. Seven weeks previously, he observed a small tumour springing up between the first and second molar teeth of the lower jaw, unattended by pain. The rapid increase of the tumour loosened the adjacent teeth, and caused them to drop out. The motions of the tongue were next impeded.

"On examining the mouth internally, I found the disease had engaged the bone from its angle to the canine tooth of the same side; the chief portion of the tumor elevating itself from the alveolar processes, inclined inwards as far as the median line: its structure was rather of a firm consistence, but capable of yielding to the pressure of the finger.

"The removal of the diseased portion of the bone having been decided upon, I proceeded to the operation on the 15th of the month. I commenced by an incision through the cheek opposite the first incisor tooth of the affected side, and divided the bone with the chain saw. The second incision was carried from the symphysis beneath the base of the jaw, to the angle. The division of the soft parts in this direction was attended with the inconvenience of wounding the facial artery, just as it turns over the jaw; its retraction under the protection of the bone subjected the patient to a greater loss of blood than could have been safely borne, had he been of a very delicate habit. The succeeding steps of the operation were conducted as in the former case, the bone being sawn through at the same points above the angle." 11.

This boy recovered without the occurrence of a single bad symptom, enjoying the power of mastication with the remainder of the jaw.

The third case is not important, and we shall pass it over.

AMPUTATION AT THE ARTICULATION.

We were rather surprised to find Mr. Cusack silent on any operation of this kind as appearing in our Journals. If he will turn to the first volume of this series, page 213, he will find Professor Lallemand's case, where the *whole* of the lower jaw was removed. In the fifth volume of the same journal, page 532, will be found an amputation of the maxilla inferior, by Dr. M'Clelland, where the whole of the bone was also removed, with the exception of one of the condyles. The Montpellier Professor performed this terrible operation long before Mr. Cusack, and the account of it in this Journal, was published *one year* before Mr. C. operated. But this is of little consequence.

*Case* 4. James Heron, aged 30, of good constitution, was received into the hospital on the 6th May, 1825. After the removal of a tooth, some months previously, a tumour sprang from the vacant space, the progress of which was slow, but ultimately extended beyond the zygoma superiorly, and occupied one half the sublingual space internally. The bone was enlarged, and the teeth loosened as far as the first incisor tooth on that side. Deglutition and articulation were much impeded, but he was free from any other distressing symptom, except a pain occasionally felt in the centre of the bone, shooting towards the ear. It was evident that the angle and the ascending branch of the bone were the parts principally engaged, and that the extent of the disease would probably require the removal of the bone at the articulation.

"On Friday, May 13, the patient being seated on a high chair, in the position already described, I commenced the operation by an incision, extending from the commissure of the lips of the affected side to the base of the bone, which was divided at the second incisor tooth: another incision, beginning at the zygoma, was carried down over the articulation, and in front of the ramus, terminating at the angle. The third, connecting the two former, passed obliquely upwards and outwards, from the termination of the first. I then dissected the cheek from the anterior surface of the tumour, which was obscured by the expansion of the masseter muscle. This being divided, the extent of the disease could be more readily ascertained. A portion of the tumor, ascending under the zygoma, completely filled up the space beneath the arch; and, from its size and position, retained the processes immoveably fixed, preventing the operator from using the jaw as a lever in the dislocation of the condyle. Under these circumstances, I decided on cutting across the ramus above the angle.

"Having accomplished this object, I removed the section of bone included between the two divisions, with the corresponding part of the tumor. As the room thus obtained enabled me to ascertain that the coronoid process was partly absorbed, and distinctly separated from the condyle, I directed my attention exclusively to the articulation.

"Mr. Colles, seizing the extremity of the ramus in a strong pair of forceps, pressed the condyle against the anterior part of the capsular ligament: by this means, the joint was penetrated with more safety and facility. I next enlarged the opening with a blunt-pointed bistory, sufficiently to allow the protrusion of the head of the bone, and the separation of its remaining connexions with the capsule, as well as the division of the attachment of the external pterygoid muscle. The operation was concluded by the removal of that portion of the tumour which was seated beneath the zygoma." 16.

No vessel except the facial artery required the ligature—the subsequent inflammation was trifling—the external wound healed by the first intention.

*Case* 5. This was a very formidable one. The patient was a pale and delicate female, 24 years of age, admitted 27th of May, 1825. She had had a tooth extracted nearly five years before, and this was succeeded by a tumour in the situation of the extracted tooth. The progress of the disease was, at first, slow; but afterwards, in the course of a year, it became visible externally. Its advancement was marked by frequent paroxysms of acute pain. In the course of the second year, the bone snapped across, about the situation of the canine tooth, in the attempt to chew a hard crust, attended with excessive pain.

The fractured extremities appear to have united, but this part became involved in the disease. During the year previous to admission, the patient was much incommoded in her deglutition, respiration, and articulation. Her health was greatly impaired—the uterine secretion suspended—appetite lost—emaciation advancing—hæmatemesis.

"On examination, I found that the tumor had engaged the entire extent of the jaw, from the articulation on the right side to the dens cuspidatus on the left; the principal bulk of the swelling extended itself laterally, inclining outwards and downwards, and distending the soft parts so much that they appeared on the point of giving way: inwards, it occupied the entire sublingual space, passing the median line, and touching the ramus on the opposite side, by which portion the tongue was elevated, and its tip brought into contact with the *velum palati*. The disease had extended to the articulation, and the coronoid process was so much expanded as completely to prevent any motion of the affected side; while the mouth, which was kept permanently open, gave passage to a continued stream of saliva. This tumor evidently differed in structure from those I had previously met with; it was more dense, the surface being smooth and yielding to the impression of the finger; but its parietes possessed so much elasticity as immediately to resume the same uniformity of aspect, upon withdrawing the pressure. The incisor teeth still remained, and in consequence of their looseness, occasioned much distress.—The general health, as I have before observed, had suffered considerably; but little nourishment could be taken, and that with much difficulty." 20.

This was, of course, a most formidable case;—the constitution broken—tumour extensive—integuments so stretched as to leave ground for apprehension that they would be unable to preserve their vitality after an operation. Nevertheless, this dernier resource was determined upon.

"On Friday, June 3d, she submitted to the operation; the preliminary arrangements for which were made in the manner before described. The mental portion of the bone was divided by the chain saw, at the situation of the first molar tooth of the left side. This step of the operation was rendered tedious by the restlessness of the patient; she depressed her head towards the sternum, and offered much resistance to the use of the saw. The transverse incision through the cheek was inclined upwards, in the direction already noticed. A third was made over the articulation, and continued downwards, to unite with the second. The division of the soft parts had been hitherto attended with a profuse hemorrhage, much exceeding what had been contemplated. The vessels, however, contracted speedily on being divided, yet the total quantity of blood lost was considerable. After the separation of the cheek, and the complete exposure of the anterior surface of the tumor, the bone was found so immoveably fixed from the expansion of the processes, that I

could not venture farther in the dissection without danger of wounding the vessels beneath the base of the jaw. In this dilemma, I divided the tumor about an inch below the articulation, and quickly separated the portion included between the two sections: ample opportunity was thus afforded for clearly ascertaining the nature of the difficulties I had still to encounter. So completely had the coronoid process filled up the infra zygomatic space, that the detachment of the insertion of the temporal muscle proved both difficult and tedious: after it had been separated, I proceeded to the removal of the condyle. Having fairly exposed the anterior surface of the articulation, I perforated the capsular ligament, and finally completed a tedious and disgusting operation, during which the patient had fainted several times, and rejected the contents of her stomach. The integuments having been replaced, and the wound dressed in the usual manner, she was removed to her ward and placed in bed; when she exhibited but feeble signs of animation, and remained in the same state of exhaustion for several hours. Her countenance was pale, skin cold, and pulse scarcely perceptible. Reaction took place but slowly;—in twenty-four hours after the operation the lip and cheek were still cold; yet, from this period, little trouble was experienced; a mild inflammatory action succeeded, and in six weeks she was restored to her friends, fat and healthy." 22.

*Case* 6. This was also a very bad case. The patient was 35 years of age, when he entered the hospital in January, 1825. In 1821, he had two teeth extracted from the lower jaw, on account of pain in the bone. That pain ceased for three months after extraction of the teeth. In 1822, a blow was received on the jaw, and the pain returned, accompanied by a very free hæmorrhage. A small tumour now arose from the place formerly occupied by the teeth. The growth of this swelling was rapid, and attended with severe pain in the bone. No remedy afforded any relief—the swelling continued to increase, and soon impeded deglutition, respiration, and speech.

" At the time of admission, the tumor had engaged the right side of the jaw; the cheek being distended to such a degree by its protrusion externally, as almost to present the appearance of a second head. The integuments had suffered much from distension, and were traversed by numerous small veins, which ramified over the cheek: internally, the tumor filled up the mouth, and displaced the tongue; passing backwards it rested on the anterior surface of the palate; and advancing forwards, protruded from between the lips, and forced the jaws to remain permanently separated." 24.

He had become greatly emaciated—had a sensation of constriction about the præcordia—and incessant cough, when he attempted the recumbent posture. The condition of the patient forbade an operation, but he was kept in the hospital with the hope that his constitution might improve under proper treatment. In three months, the integuments over the most projecting part of the tumour sloughed to the extent of a crown-piece, leaving an opening which continued gradually to enlarge, and through which a mass of soft florid granulations protruded, and, after a time, sloughed away also, to be succeeded by a new series of the same, their structure highly vascular, irritable, and hæmorrhagic. His health remained stationary through the summer; but the local disease advanced along the bone. In the month of September, a decided amelioration of the general health took place, contrary to expectation. His flesh increased —his appetite improved—the sense of constriction about the præcordia disappeared—and, in this favourable condition, an operation was decided on.

"The position of the patient, and all the preliminary arrangements, being disposed exactly as in the preceding cases, I commenced the operation by an incision, which connected the commissure of the lips of the affected side to the nearest point of the newly formed opening in the cheek. A second incision descended along the inner border of the opening. A third was prolonged in the direction of the symphysis, towards the left side, as far as the second molar tooth, which had been previously extracted. I next dissected off the flap thus marked out. The instant the separation of the under lip was completed, a large mass of the tumor, which had been supported and retained by this barrier, burst forth, presenting a congeries of fungous or granular masses, which separated into distinct portions: the sight was so appalling and unexpected, that many present were alarmed for the result. It was, however, too late to recede: I directly applied the chain saw through the alveolar process, from which the tooth had been removed. I next circumscribed the chasm in the cheek, by two incisions, which were extended outwards, and united over the external side of the tumor; another incision being made in the direction of the condyloid cavity, and prolonged downwards to the point of union.

"I then proceeded to expose the anterior surface of the tumor, when upon minute examination, it was found that no trace of the right side of the jaw bone existed; the whole having degenerated into a softened structure, separated into distinct portions by deep fissures. To insulate the entire disease at once was impossible; I, therefore, removed the mental portion in the first instance; and by a little care, was able to accomplish the separation of the entire mass, although broken up into several pieces: in this part of the operation, the cutting edge of the knife was used as little as possible. The salivary glands were completely laid bare, being exposed to view with all the vessels and nerves which lie under the angle and base of the jaw.

"These different parts, with the surrounding structures, were ascertained to be perfectly healthy. The diseased mass rested in a bed of cellular substance, which greatly facilitated its removal. It was a curious fact, that no trace of either of the processes could be observed: indeed, so perfect was the disorganization, that only about one inch of *bone* was obliged to be removed. The quantity of blood lost during the operation was not very considerable, the few vessels that required a ligature being readily secured. The chasm which remained was filled up with lint; the edges of the wound approximated, and retained by points of interrupted suture. After having been placed in bed, the patient was somewhat feeble, and continued weak for several hours; but was, in a short time, restored by the use of some simple cordials." 29.

After this formidable and bold operation, the man's health rapidly improved—he was soon able to leave his bed—the wounds quickly healed—and he continues to enjoy good health.

The seventh case we have not space to describe. The whole of the lower jaw bone was removed, and the patient was doing well till the sixth day, when erysipelas supervened, and she died on the tenth day from the operation.

The paper concludes with some general directions for conducting this operation, for which we must refer to the volume. Mr. Cusack has done great honour to British surgery by these daring and successful operations, and his character must always stand high in the first rank of modern surgeons.

### Art. II.

*Report of an Inquiry into the Value of Mediate Auscultation, as a Method of Diagnosis in Inflammations of the Pleura, Lungs, and Bronchia.* By William Stack, M.D. Physician to Sir P. Dun's Hospital.

Auscultation is making rapid advances in the Sister Kingdom—and the value of it, as a means of diagnosis, is not now attempted to be questioned, except in holes and corners, by old drivellers who have gone past the period of learning any thing, or by lazy routinists who will not take the trouble of increasing their knowledge, which, indeed, they think is quite unnecessary. The author of the above extended paper is not, however, inclined to assert that, by means of auscultation and percussion, we can always distinguish between inflammation of the mucous, serous, and parenchymatic structures of the thoracic viscera, in acute inflammation of these parts, and in an early stage, but the case is very different when the disease has been somewhat protracted. Thus, a patient presents himself with dyspnœa which has lasted for several days, having been preceded by pain in some part of the chest. On percussion one side returns a dull sound, but does not appear either dilated or contracted. On examination with the stethoscope, the respiratory murmur is absent all over that side of the chest, with the exception, perhaps, of those parts where the root of the lungs is situated, and a few inches down the spine. There is neither bronchophony (except in the superior parts) nor ægophony. What is to be the diagnosis? Is the disease pneumonia or pleuritis?

"I say it is impossible to determine, even supposing the minor signs to be present, such, for example, as the communication of the heart's action over the affected side, which some will have to be a sign of the advanced stage of pneumonia, but which may also depend on other causes, such as dilatation with hypertrophia of the heart. The disease may be either acute pneumonia or acute pleuritis, or it may be chronic pneumonia or chronic pleuritis.

"It is true that the nature of the sputa may afford us assistance in this case, but it must always be remembered that the appearance of the expectoration is more variable in chronic than in acute inflammations; and that both in chronic pneumonia, and chronic pleuritis, it very frequently assumes the form of the expectoration of catarrh." 93.

The following case is adduced in illustration. We shall considerably abridge the narrative.

A man was admitted into hospital on the 26th May, who had been seized 14 days previously with sense of oppression and tightness in the left side of the thorax. He had slight pain in the left side, but not, at that time, so as to impede inspiration, or prevent lying on either side with ease. Latterly he could rest on the left side better than on the right. He was bled at the commencement, and had little or no expectoration —but at present it is abundant and catarrhal.

"Exploration of the chest gave the following result: no dilatation or contraction of the affected side; dull sound on percussion over the entire of that side; no respiratory sound whatever on the anterior part, except just at the junction of the clavicle with the sternum when there was bronchial respiration; no ægophony, no bronchophony. On the posterior part there was an indistinct respiratory murmur down the spine to the eighth rib. The alteration of the position of the patient produced no change in the results obtained. On the 17th, the respiratory murmur was faintly audible on the anterior of the thorax, on a level with and below the apex of the heart; no ægophony; the sound on percussion less dull than at the superior parts. On the 20th, the auscultatory signs were the same as on the 17th, but the expectoration had been presenting various appearances since his admission; sometimes it was liquid, transparent, ropy, and covered with a frothy surface; sometimes more dense, opaque, and inclining to a greenish cast; occasionally (as on this day) approximating to the rusty colour of pneumonic sputa. 21st, Bronchophonia in several places down the spine, along the clavicle and spine of scapula, and those places where it is generally audible in health. The sound, however, not so remarkable as at the healthy side; he now lies on either side, or on his back, with perfect ease. In a few days he left the house, having no complaint and the respiratory murmur becoming daily more distinct." 94.

This case was treated as one of pleuritis, effusion being supposed to have taken place before he entered the hospital. But the auscultic signs were equally indicative of pneumonia or pleuritis. One of them, indeed, (the return of the respiratory sound at the *inferior part)* seemed rather to point to pneumonia than pleuritis. Dr. S. has not taken into the account the possibility of the disease being bronchitis, because it very rarely happens, though not always, that in this disease the chest returns a dull sound on percussion. An interesting exception to this general rule is related at page 96 of the volume. The bronchial inflammation had been very acute and rapid, producing a state approximating to asphyxia, while the effusion of fluids into the various branches of the bronchia and air-cells rendered the lungs so dense as to communicate the shock of the heart's action to all parts of the chest, as in hepatization, depriving the lung, at the same time, of all respiratory murmur, or sonorous percussion. On dissection, no inflammation whatever was found in the substance of the lungs, but the air-passages were charged with viscid fluid. Of all diagnostic signs in inflammatory affections of the thoracic viscera, Dr. S. depends most on the *colour of the countenance.*

"Each of the three inflammations, I speak of the acute forms, pleuritis, pneumonia and bronchitis, possesses, when the disease is at all severe, the characteristic of a peculiar colour of the countenance. In pleuritis it is either of a flushed and florid, or of a natural hue. In humid* bronchitis it is of a colour more or less approaching to a blue, according to the severity and extent of the inflammation. In pleuritis the lips are of a florid red; in humid bronchitis they are of a blue colour; the flush of pneumonia is altogether different from either; it is, as it were, made up by an intermixture of the two shades, but quite distinct from each; it is also less remarkable in particular isolated parts than the colour arising from humid bronchitis and pleuritis; in both these the cheeks are often flushed in a circumscribed manner, the lips are always remarkably coloured; but in pneumonia the lips and cheeks are scarcely more suffused than the adjacent parts, and the flush, though sometimes circumscribed, appears more generally diffused. It seems to me not very difficult to account for this variety of colour in these different diseases; in acute bronchitis, especially where it prevails epidemically, the affection is commonly of a generally diffused nature, and extends over a considerable portion of the lungs (at least in this country,

* "I use the term humid for the purpose of shewing that these observations apply to that form of bronchitis, (which is by far the most frequent) in which an increased secretion takes place from the mucous membrane."

although from the clinical records in other places the contrary seems to be the case;) the nature of the morbid change is such as to afford a very considerable obstruction to the transformation of the venous into arterial blood; the consequence is, that blood very little differing from venous is circulating in the arterial system; this easily accounts for the bluish cast of the countenance. In pneumonia the affection is not, in general, so universal, and in but comparatively few instances does it happen that both lungs are engaged; in either case the progress of the complaint is not so rapid as to afford an instantaneous impediment in the affected part to the act of hematosis; but the blood which circulated in the pulmonary artery and veins through the affected part is, up to a certain stage of the disease, partially converted into arterial blood; this is poured into the left side of the heart, and there mingled with the perfectly formed arterial blood coming from the sound portion of the lungs, producing but a slight variation from the colour of true arterial blood. It is obvious, therefore, that except in cases of a violent nature, or where the inflammation occupies a considerable extent of lung, the alteration which the colour of the countenance undergoes must be but slight, and that this alteration is such as would arise from the circulation of blood not bluish, as in the case of bronchitis, but rather of a dull red, or brick colour. In pleuritis, while the disease occupies one side only, scarcely any obstruction is offered to the transformation of venous into arterial blood; for the pulmonary tissue and mucous membrane being unaffected, the process of hematosis will be perfected in all the parts of the lungs which are permeable by the air. If indeed the affection be general over one side, or if the inflammation be so violent that a copious effusion is suddenly produced, then it may happen that the lung will be compressed or folded on itself towards the root, and remain nearly inoperative in that process; but still all the blood which *is* transmitted through this lung will be properly transformed, and so no blood can pass to the left side of the heart but such as possesses the genuine florid colour of arterial blood. If therefore there were no accompanying fever, the countenance would remain, as far as colour is concerned, in a perfectly natural state; but inasmuch as there generally is a considerable degree of concomitant fever, and that too of the angiotenic form, (that is, where the circulating system is chiefly engaged,) it is evident that the capillary system must be injected with a florid blood, and so produce that floridness of countenance which is peculiarly remarkable in cases of violent pleuritis. If both sides are simultaneously affected, a case which rarely occurs, then the obstacle opposed by the pleuritic effusion to the dilatation of both lungs causes a very material obstruction to the transformation of the venous into arterial blood, and blood will pass to the left side of the heart, notwithstanding the compressed state of the lungs, which has not been completely submitted to the action of the atmosphere; the countenance therefore will assume an appearance more or less similar to that of pneumonia or bronchitis, according to the severity of the complaint. What I have now said applies to these affections only when in an uncomplicated form; when they are complicated with each other the flush peculiar to each is of course modified by that attendant on the superadded affection; for example, in pleuro-pneumonia, (that is, where inflammation of the parenchyma of the lungs is complicated with pleuritis,) we may expect to find the dusky lurid red of pneumonia assuming the place of the florid cast of pleuritis, because a moderate degree of inflammation of the lung will produce a greater deviation from the natural color of the countenance, than the same degree of inflammation of the pleura." 101.

The above passage contains interesting observations. In the succeeding pages, Dr. S. enters into a rather too minute inquiry respecting the priority of inflammation in one or other of the tissues above-mentioned. We cannot follow him in this investigation, but refer those who are curious on this subject to the volume itself.

### Art. III.

*Cases of Excision of Carious Joints.* By Philip Crampton, F.R.S. Surgeon-General in Ireland, &c.

The surgical reader is aware that Mr. Park, of Liverpool, long ago extirpated the whole of the knee-joint, in a case of what is called white swelling, removing somewhat, though not much more than two inches of the femur; and of the tibia rather more than an inch. The patient lived to make several voyages to sea afterwards, and was able to go aloft with agility. A few years after the publication of Mr. Park's case, Moreau the younger published "Cases of Excision of Carious Joints," which were violently opposed by the surgeons of that day in France. Since that period, the operation seems to have attracted but little notice, and we are not aware that any cases have been made public, where the operation was repeated, before the present paper. We shall, therefore, proceed at once to an analysis of these cases, three in number.

*Case* 1. A. Gordon, of the 90th Regt. aged 23, was admitted into the Royal Infirmary on the 2d Jan. 1823. The scrofulous aspect in this patient was most decided and unequivocal, and well-marked white swelling of the right elbow-joint, of ten months standing. The swelling extended full a hand's breadth above and below the joint—suppuration had taken place over the inner condyle of the humerus, and the opening had degenerated into a large and irregular ulcer, at the bottom of which the bone could be felt carious. The general health was much impaired—pulse 120, and feeble—nocturnal perspirations—in short, hectic fever was established. Consent was obtained for excision of the joint, which was performed on the 4th February, in the presence of numerous surgeons, civil and military.

"The patient was placed (as recommended by M. Moreau) upon his belly on a table covered with a mattress, and pillows so arranged, as to make his posture as little inconvenient as possible; the diseased arm hung over the edge of the table, presenting its posterior and inner surface to the operator; the brachial artery being compressed by an assistant, an incision was now made along the spine of the inner condyle, commencing about four inches above, and terminating about two inches below, its tuberosity. This incision passed through the centre of the ulceration, and laid bare the ulnar nerve, which was carefully raised from its groove, and drawn to the inner side of the incision.* A similar incision, parallel to the first, was made on the outer side of the humerus, and then a transverse section, which cut through the tendon of the triceps muscle, immediately above its insertion into the olecranon, connected the two longitudinal incisions, so that the wound represented pretty accurately the letter H; the lateral incisions, however, being slightly incurvated, so as to follow the bend which the fore-arm made with the arm. The upper flap, consisting of the lower extremity of the triceps muscle, the thickened and diseased cellular substance, and integuments, was raised from the flat surface of the humerus, to which it had a very slight attachment; the lower flap was separated in the same manner, so as to lay bare the upper extremity of the ulna and radius; the scalpel laid on its flat was now pushed between the flexor muscles and the bone on its anterior surface, at the distance of three inches above the tuberosity of the inner condyle, and retained in this situation by an assistant. The saw was then applied, and the bone was divided immediately over the flat surface of the knife, which served as a protection to the muscles beneath. The separated portion of the humerus was now raised with the utmost ease by the finger and thumb of the left hand, while the capsular and lateral ligaments, degenerated to the state of a lax cellular substance, were separated by running the knife round the condyles, keeping the edge as closely as possible to the bone. The lower extremity of the humerus being removed, the articulating surfaces of the radius and ulna were completely exposed; but, with the exception of the cartilage which covers the olecranon, (which was partially eroded,) every thing appeared sound. The olecranon was now removed, and the wound was spunged out; as there was no bleeding which rendered it necessary to have recourse to a ligature, the flaps were laid down, and secured to each other by four points of suture. The fore-arm was placed at a right angle with the arm; the wound was covered with pledgets of lint, wetted with spirits and water, and the man was laid in bed, with the arm supported on a suitable pillow." 194.

---

"* From neglecting this precaution in M. Moreau's case, the ulnar nerve was cut across, and the ring and little finger were deprived of the powers of motion."

Suppuration, attended with mild symptomatic fever, was established on the fourth day—by the ninth day he was able to sit up in his chair—and a few months at the sea-side produced a complete recovery. He was examined, on the 18th September, by a Board of General Officers, when the following was found to be the state of the case.

"At this time, the wound, with the exception of a small superficial ulceration about the place which had been occupied by the inner condyle, was completely closed; the arm, when allowed to hang by the side, retained nearly a semi-flexed position, but by a voluntary effort he was able to give a slight degree of flexion to the fore-arm, so as to lessen the angle which it formed with the arm. He had the use of the fingers, so as to be able to use his knife and spoon; and on the 27th of Nov. 1823, *he signed his own discharge with the right hand.*" 195.

The success of the above operation induced Mr. Crampton to undertake a second on the 7th of May, 1823, of which we shall here give some account.

*Case* 2. Susan Conolly, aged 23 years, of a strumous habit, and emaciated appearance, presented herself with the right knee considerably enlarged, of an irregular shape, projecting over the tibia, on the inner side, and measuring three inches and a half more than the sound knee. Severe pain is caused by the least pressure or motion, and there is a free discharge of thin, greenish-coloured matter from an ulceration under the inner hamstring. The joint was permanently contracted to a very acute angle with the thigh—febrile symptoms, with diarrhœa. The disease had commenced twelve months previously—the contraction of the joint, severe pain, and alteration in the health, were of only six months' standing. She has had regular attacks of hectic fever, with profuse perspiration and diarrhœa, for six weeks past. The operation was performed in the following manner.

"An incision, commencing about three inches above the outer condyle, and a little below the axis of the femur, was continued to about an inch below the head of the fibula. The acute angle, which the leg formed with the thigh, necessarily gave to this incision the form of a crescent. In making the incision the knife was carried down to the bone; a similar incision was made on the inner side of the joint. The lateral incisions were united by a transverse cut carried below the patella. The flap, thus formed, was raised by a rapid dissection, and the cavity of the joint was completely exposed: for the extent of more than three inches above the condyles the femur was without periosteum, the purulent matter lying in contact with the naked bone. At the point where the periosteum appeared to be united with the bone, the saw was applied, and the bone was divided, the soft parts being protected by a spatula which was passed between the muscles and the bone. The separated portion of the femur was now dissected out, and so slight were its connexions with the soft parts, that this part of the operation, which I expected would have been attended with some difficulty, was effected with the greatest ease. The articulating surface of the tibia was now fully exposed; it was totally deprived of cartilage, and was in a state of caries. By means of a strong and short knife, such as is used by shoemakers, I was enabled to pare away about half an inch of the head of the tibia, the cancelli of which were loaded with a lardaceous matter, and with pus.

"The cavity of this great wound was now sponged out, when, upon minutely examining the cut surface of the Femur, I found that the cancelli were diseased and filled with pus, and posteriorly the periosteum was detached from the bone. I therefore sawed off about an inch and a quarter more of the femur. On placing the extremities of the femur and tibia in contact, the flap, containing the patella, was found to be about three inches too long, and as the patella itself was totally deprived of its cartilage, and in a state of caries; the exceeding portion of the flap, including the patella, was removed by a transverse incision. No artery was divided which required the application of a ligature. The flap was retained in its position by two points of the interrupted suture, and compresses, wetted in spirits and water, were laid over the wound. The limb was now placed in position in one of Asselini's "carrying splints," which had previously been carefully adapted to the size and length of the limb, it extended from above the trochanter major on the outside, and from the ramus of the pubes on the inside, to about four inches below the foot, it was supplied with a sole piece, which supported the foot, and was carefully padded with a mixture of baked hair, and wool." 199.

The poor woman bore the operation with great fortitude, and Mr. C. thinks it probable that the pain was not greater than in a common amputation. The operation was succeeded by very little constitutional disturbance, and, curious to relate, this great wound united by the first intention, and was healed in less than three weeks. She continued to mend in health until the September following, when she was seized with erysipelas of the leg and thigh. The erysipelas was succeeded by abscess, which burst through the old sinus in the ham, and continued to discharge for three or four weeks, and then healed. In the month of November, she was able to go about the hospital, the limb being supported by a splint, constructed so as throw the weight of the body on the tuberosity of the ischium. She was discharged on the 27th June, 1824, in very good health; but no union had taken place between the femur and tibia. She died three years and two months after the operation, of disease of the lungs.

Although this operation completely failed, as far as the use of the limb was concerned, yet it proved that so large an articulation as the knee-joint might be excised with safety, and gave strong grounds to hope that, under more favorable circumstances, the operation might be attended with success. Indeed, it is evident that excision was not applicable to this case; for the posterior part of the femur, above the condyles, was in a state of necrosis, being deprived of periosteum, and as thin as card-paper. The disease had proceeded too far; and could the whole of the diseased bone have been removed, and union taken place, the limb would have been too short to be of any use.

*Case* 3. Ann Lynch, aged 23, a strong and healthy-looking country-girl, was admitted on the 3d of May, 1823. About four years previously, she was seized with severe pain in the right arm, which shifted to, and settled in the right knee. The pain continued more or less severe, and principally confined to the inner condyle, increased by pressure or motion. The joint became perfectly stiff, and a good deal contracted—the swelling gradually increased, until it attained a large globular form, elastic to the touch, of a dusky red colour on the inner side, and intersected by numerous large blue veins. The pains were excruciating, especially at night, and latterly symptoms of approaching hectic manifested themselves. The treatment, both local and general, employed at the hospital, contributed much to the amelioration of her general health, and, as she was anxious to undergo the same operation as the last patient underwent, it was performed on the 4th of August. But, although she manifested great courage and resolution previously, yet, from the moment the knife was applied, she lost all fortitude, patience, and, it might be said, reason itself. The most terrible difficulties were experienced by the operator, and four men were unable to hold her in any degree quiet on the operating-table! With all these disadvantages, the joint was excised, as in the case of the preceding patient. The patella, which was found to be carious, was removed with the lower portion of the flap; but the articulating cartilage of the tibia appearing sound, the greater part of it was pared away, and the semilunar cartilages were removed. The treatment was conducted on the same principles as in Conolly's case, but great difficulty was experienced in securing proper position, on account of the patient's restless disposition. The extremity of the femur was often protruded through the wound. The general health was but little affected by this formidable operation—a small exfoliation took place from the femur—and, in two months, she was removed from her bed to a chair. In about four weeks after the exfoliation of the femur, the wound was completely healed, and the limb had acquired a considerable degree of firmness. In six months, the femur and tibia were consolidated by a firm, bony union—she first went on crutches—and afterwards was able to walk several miles in the day—in fact, to use her own words, she "was able to stand or walk the length of the day."

We have not space to give any account of the observations and reflections which these operations have given rise to in the mind of Mr. Crampton. For these we must refer to the volume itself. The intrepidity, judgment, and dexterity of Mr. Crampton are well known, and these operations do not tend to diminish their lustre.

### ART IV.

*Second Communication relative to the fatal Consequences which result from slight Wounds received in Dissection.* BY A. COLLES, M.D.

Of Mr. Colles's first communication on this melancholy and interesting subject, we gave an ample account in a former number of this Journal. We shall now proceed, at once, to the present paper.

*Case.* On Tuesday, 18th of May, Mr. Shekelton examined the body of a man who had died of peritoneal inflammation consequent on lithotomy. The examination took place in a few hours after death, the body still retaining its heat. Mr. S. pricked his finger, which called forth the usual expression of pain, but it was not further attended to, the dissector continuing to immerse his hands in the contents of the pelvis for a considerable time. *Wednesday*, 19*th*. He felt unwell. 20*th*. Gave a demonstration, and immediately afterwards went home very ill, but did not apply to his medical friends. *Friday*, 21*st*. Mr. Cusack accidentally saw him, and found some of the left axillary glands swelled, from which he augured no good. 22*d*. Mr. C. again saw Mr. S. and found the patient's condition improved. 23*d*. Mr. Colles saw the patient, who observed emphatically that he had spent a wretched night:—yet the febrile symptoms were not severe—pulse 84—skin hot—indescribable anxiety and distress—feeling of fulness in the stomach—glands in the axilla slightly enlarged and tender to the touch—no swelling or redness along the arm—pain along the course of the ulnar nerve and down the left side of the thorax—no appearance of inflammation in the ring-finger which had been wounded—indeed there was scarcely any trace of wound. By the patient's own desire a few leeches were applied to the axillary gland—he took an emetic, which operated well, but made no impression on the symptoms. 24*th.* Uneasiness in the stomach and bowels—tongue coated—complexion assuming a yellow tinge—features rather sharp—no uneasiness in the course of the lymphatics of the arm—cessation of pain and swelling in the glands—hope indulged that the case would only turn out to be one of common fever. 25*th.* Constitutional distress increased, and the real nature of the disease too obvious. A full opiate at night. In the evening a red spot, the size of a shilling, appeared on the right patella, attended, for two hours, by the most severe torture in the knee, referred to the cavity of the joint. 26*th.* Two doses of laudanum last night produced profuse general perspiration, but no alleviation of the symptoms. He declared he had passed a most uncomfortable night. In the course of the night, his brother (a physician) observed a large red and swollen patch over the right tibialis anticus. This and the spot on the patella had a solid feel, but were not very tender to the touch. At 2 A.M. this morning (26*th*) the right arm, from the shoulder to the elbow, was observed to be swelled, though not discoloured. The right thigh was also in the same state. A red patch was seen on the dorsum of the left foot—pain complained of in the left scapula and shoulder—tongue thickly coated—countenance of a deep yellow tinge. 27*th.* Pain in the left arm, with considerable swelling—universal yellowness of the skin. In the evening extreme weakness. 28*th.* At five this morning he sent for Mr. Colles, and requested him to make incisions into the left arm. Mr. C. could not refuse to comply with the patient's wishes, though he knew the operation would be unavailing, as the hand was livid and cold—all the extremities cold. Several incisions were made, but nothing except a small quantity of blood issued. He died at nine o'clock that morning. *No dissection.*

In this case, our author observes, we recognise the same train of symptoms as in the cases of Mr. Hutchinson and Mr. Dease, reported in the third volume of the Dublin Hospital Reports.

"The present case tends to strengthen the opinion I then advanced, 'that slight wounds received in dissecting fresh bodies, sometimes give rise to a peculiar disease, perfectly distinct from every other disease consequent on similar wounds.' In addition to the other symptoms there described as being characteristic of this disease, we may mention this striking one, viz. that previously to the disease terminating either in death or in recovery, swelling and inflammation seize upon the portion of the limb interposed between the original wound and the first seat of pain. We see that this took place in Mr. Hutchinson's case at so late a period as three weeks; the fever, although it had remitted, not having ceased until this inflammation had occurred. In the fatal cases of Mr. Dease and Mr. Shekelton it appeared, in the former on the 9th, in the latter on the 10th day.

"I must entreat the attention of the reader to this fact, that the redness which is seen on the swollen parts is very unlike to that of erysipelas, for the colour is that of a peach-blossom, is of very small extent compared with the extent of the swelling, is seen for a few days, perhaps for a few hours only, on the same spot, and next is observed in some very distant part, possibly in the opposite limb; besides, this peculiar redness vanishing quickly from a part, does not leave any vesication or desquamation after it, as is seen in cases of erysipelas.

"If any proof be wanting to establish an essential difference between this disease and phlegmonoid erysipelas, it will be found in the state of the swollen parts when cut into. For although four incisions were made into different parts of the left arm of Mr. Shekelton, yet no discharge, except a small quantity of blood issued, nor was any change of structure visible except that slight one which has been noticed at the bend of the arm.

"This sudden shifting of the swelling and redness from one to another, and very distant part, is not to be confounded with what we sometimes observe in chronic diseases, viz. an abscess suddenly appearing in a part remote from the original seat of the disease. For in such cases the abscess quickly forms, and with this peculiarity, that we feel the fluctuation of pus although we have scarcely any marks of preceding or accompanying inflammation: the skin is not reddened until the ulcerative process is about to give exit to the fluid. In such cases, although we may have a succession of abscesses, yet we have not any instance of a sudden swelling accompanied by a light blush of redness, and of its equally sudden disappearance, leaving after it only a slight degree of swelling, without any other symptom or trace of inflammation.

"In short, this peculiar disease, the effect of slight wounds received in dissection, presents much less of inflammation of the wound or its vicinity than occurs in the various other diseases to which slight injuries more frequently give rise. Here it seems to produce mischief by exciting a fever, which in its turn induces a swelling and redness of very peculiar characters, although at length (if the patient chance to survive) it will end in inflammation and suppuration of the wounded limb." 248.

Whatever opinions may be entertained respecting the nature of the disease, there can be but one respecting its fatality under every mode of treatment which has yet been devised. The following suggestion of a man who has had much experience in this direful malady, is deserving of great attention.

"The plan which I would suggest as most likely to succeed (for as yet it is untried), is to administer calomel with the view of speedily exciting ptyalism. Many experienced practitioners I know are in the habit of combining this medicine with opium, when their object is to excite ptyalism very quickly. But I should prefer giving it in an uncombined form, and in doses of three grains every three or four hours. When administered in this manner, it seldom fails to produce salivation in thirty-six or forty-eight hours, provided that the two or three first doses affect the bowels. In cases where the bowels are not moved by the first doses of this medicine, it will be necessary, in order to ensure its effects, either to combine the calomel with some purgative, or occasionally to interpose a purgative. When thus administered it will very seldom disappoint our expectations. Mercury administered in other forms of fever has so often succeeded in effecting a cure, that I think we may, with some confidence, anticipate its good effects when administered in the fever consequent on wounds received in the dissection of very fresh human bodies." 249.

### Art. V.

*A selection of Cases from the Medical Wards of the Meath Hospital and County of Dublin Infirmary.* By R. J. Graves, M.D. and William Stokes, M.D. Physicians to that Institution.

The principal object of this report is to exhibit the histories and dissections (where dissections were made) of cases proving the utility of the stethoscope in the diagnosis and treatment of thoracic diseases. This paper will shew that auscultation is much more diligently, and successfully cultivated in the wards of the Irish than of the English hospitals. Into the reasons for this difference we shall not, at present, stop to inquire; but proceed to the cases themselves.

*Case* 1. J. Connor, aged 33, was admitted into the hospital for dysentery, 13th September. His stools were bloody—he had tenesmus—prolapsus ani. Calomel and opium were given till salivation was established, when some relief was obtained. Small doses of turpentine completed the cure of dysentery; but the lower extremities began to swell early in October, for which colchicum was given with advantage. On the 13th October he was seized with orthopnœa. It was suspected that he might have pneumonia, but the diagnosis was rendered doubtful by the sudden occurrence of the pectoral symptoms after the disappearance of external dropsy in a debilitated constitution. The diagnosis between pneumonia and hydrothorax was a matter of great importance in this case, and "it was at once afforded by an examination with the stethoscope." It was observed that he lay on the left side. Here the crepitating râle was heard above the left mamma—the respiratory murmur being inaudible beneath, pulse 105. "*Diagnos. inflammation of the lower part of the left lung.*" Bled to xiv. ounces. This was followed by the most beneficial effects. The orthopnœa, cough, and pain of the side disappeared—he slept well—and the pulse fell to 95—respiration tranquil—respiratory murmur audible under the left mamma, but weaker than natural—crepitus has disappeared. He got considerably better, and, on the 28th was pronounced convalescent; but was suddenly seized again, in the night, with dyspnœa, heaving of the chest, hard frequent cough without expectoration. He could only lie on the left side—pulse full and strong, at 108. *Auscultation, respir. murm. indistinct below the left mamma—crepitat. râle under the left scapula.* Bleeding and blistering relieved the above symptoms, and dissipated the crepitus. On the 3d November, he was again attacked by constant hard cough. Percussion over the inferior part of the left lung gives a dull sound, and the crepitus was again heard there. The impulse of the heart was much stronger than natural, and the sound is heard over the whole of the right side. The sound of the right ventricle is much clearer than that of the left, which is dull and prolonged. The auricles give a clear sound. There is no induration of disease of the valves. It was conjectured, from these data, that there existed active aneurism of the right ventricle. The respiratory murmur gradually diminished over the anterior part of the lungs; and, from this time, no accurate examination by the stethoscope was made. He sunk with symptoms of effusion in the chest, on the 17th December.

"*Dissection.*—Much serum in the cavities of the thorax. The lower lobes of the right lung are hepatized, soft and of a red colour; the superior lobe is inflamed in the first degree. The lower lobe of the left lung, especially in the point corresponding to the left mamma, is exceedingly solid and of a very dark grey colour.

Active aneurism of the right ventricle; the left is greatly thickened without alteration of its capacity. No disease of the valves, but there is a considerable thickening of the aorta, forming a semi-cartilaginous ring immediately above its origin, and causing a diminution of its calibre. The lining membrane of the aorta is much thickened, and apparently tuberculated. This appearance extends for several inches, and is caused by smaller depositions of cartilaginous matter." 73.

Our authors think themselves justified in concluding that this man's life was prolonged by the stethoscope. "He was attacked with all the symptoms of hydrothorax, but the cylinder shewed that his complaint was pneumonia, which would have speedily cut him off, had it not been checked by the lancet." The disease returned four different times in the course of two months, and at length carried off the patient. "Its situation, its intensity, and the effects of remedies upon it, were, in every instance, pointed out by the stethoscope with the utmost accuracy, as was proved by the sectio cadaveris."

*Case* 2. *Phthisis.* Anne Kelly, aged 27, was admitted early in November, with all the symptoms of phthisis. Her expectoration was copious, consisting of transparent mucus, albuminous looking masses, and puriform striæ. Lies always on the right side. Complaint of five months standing.

" *Auscultation.*—On the right side, about two inches below the clavicle, the voice appears to issue from the stethoscope in the most distinct manner; and is perfectly articulate, and the last letter of the concluding word is very distinct. In other words there is true pectoriloquism. At this spot the respiration is distinctly cavernous, giving the idea of air entering a cavity in the pulmonary tissue. At the antero-inferior part of the left lung there is bronchial respiration of the most evident kind; the sound is sharp, and the air appears to be drawn in through the stethoscope. Crepitating râle over the remaining portion of this lung. The sound of the heart is heard in its superior portion." 75.

*Diagnosis.—A tubercular excavation in the middle lobe of the right lung; solidification of the superior part of the left lung; inflammation in the first degree, in the remaining portion.*

On the 9th November the bronchial respiration and crepitus had disappeared. *Diagnosis.* The left lung has become solid. We need not pursue farther the auscultation and diagnoses. She lingered until the 27th of December, when death terminated her sufferings.

" *Dissection.*—The lungs were universally adherent to the cavity of the chest. In the inferior part of the middle lobe, on the right side, we found an excavation with firm parietes, and about the size of a large walnut. The superior lobe was found also excavated in a more irregular manner. The remainder of the lung was studded with tubercles;—the lower lobe had much fewer of them, was still crepitating, but appeared engorged with a frothy and sanguinolent mucus.

The left lung appeared completely solid, but on cutting into its substance, an extensive tuberculous excavation was found capable of containing two oranges. This communicated with many other smaller ones by winding canals; and in the inferior portion of the lung another excavation of great size was found." 77.

This case, they properly observe, is very interesting, inasmuch as the entire train of pathological phenomena was traced by means of auscultation. Pectoriloquism indicated a cavity above the right mamma—the crepitating râle heard over two-thirds of the left lung, indicated that inflammation was going on—while the bronchial respiration shewed that the upper part of the lung had become solid.

*Case* 3. *Emphysema of the Lung.* Daniel Grogan, aged 40, came into the hospital affected with cough, dyspnœa, general anasarca, and livor of the countenance, with paroxysms of orthopnœa during the night—expectoration free, and like the white of eggs—constantly lies on the left side, which presents nothing remarkable in form; but on the anterior part of the right side there is a remarkable convexity or prominence, extending from the middle of the third to that of the seventh rib. States that he has been subject to asthmatic cough from boyhood—but that, in the preceding September, he became anasarcous of the lower extremities, after which he caught cold, and the present symptoms came on. Pulse 110, small and equal. Chest sounds very hollow on percussion, especially in the situation of the prominence—posteriorly the sound is not so clear. The respiratory murmur is almost inaudible over the anterior part of the thorax. A hissing râle is heard at intervals over the whole thorax, especially when he takes in a deep inspiration—posteriorly it is mixed with the sonorous râle. Sound and impulse of the heart are heard and felt over the whole of the right side—strong pulsation at the ensiform cartilage. The following diagnosis was entered on the books. "*No hydrothorax—emphysema of the lung—bronchitis—hypertrophy of the heart.*"

On the 15th January, all these symptoms were increased. Below the scapula, the sound, on percussion, had become dull, and, on applying the cylinder, a strong crepitating râle was heard. The voice resounded under the stethoscope; but the words were not articulate. *Diag. inflammation of the posterior part of the left lung.* Venesect. ad ℥ xij. and to take purgative medicine. By these he was much relieved. On the 18th, the strong impulse felt at the superior part of the right side, corresponding with the ventricular contraction, led them to suspect aneurism of the aorta. There was none of the rustling sound termed "*bruit de soufflet,*" to be heard. He died on the 29th. Before dissection, the following diagnosis was written down.

"Emphysema, particularly of the right lung; bronchitis, entire enlargement of the right side of heart, *perhaps* of the aorta. *Remains of pneumonia* in the posterior part of the left lung. Hydrothorax may have taken place immediately before death.

"*Dissection.* Upon raising the sternum the lungs did not collapse, but appeared firmly bound down by adhesions so universal, that the cavities of the pleuræ were completely obliterated, thus preventing the possibility of effusion. In both lungs the lobes were united, but this union must have been the consequence of recent inflammation, as the coagulable lymph thrown out was soft, and the interlobular pleura beautifully injected with red vessels. The adhesions between the pulmonary and costal pleuræ, on the contrary, appeared to be the consequence of a former affection, as they were exceedingly strong, and on the antero-superior part of the right lung the membranes were converted into a thick white and cartilaginous substance. The whole of the right lung was in a state of emphysema, all the air cells appearing dilated, and the pleura in many places raised into vesicles the size of a walnut; when cut into, these vesicles were found divided by membranous septa, perpendicular to the surface of the lung. The volume of this lung was double that of the left, its bronchial tubes filled with a muco-purulent fluid, and their lining membrane of a bright red colour. The left lung was much diminished in size, the upper part covered with large vesicles, the lower of a pale colour and flabby consistence, but still presenting the dilated air cells. Upon cutting into this portion of the lung we thought the knife had entered an abscess, as a large quantity of a viscid and yellowish fluid flowed out and displayed a cavity in the pulmonary tissue, capable of containing a moderately sized apple; but on closer examination this cavity proved to be an enormously dilated bronchial tube, as it was lined by a delicate mucous membrane, continuous with that of the bronchial tubes, and beneath which traces of the cartilaginous rings, peculiar to these canals, could be observed. All the bronchial tubes on this side were thus more or less affected, so that the lung appeared to contain many small abscesses. Posteriorly the pulmonary tissue was of a dark grey colour, and cartilaginous hardness, evidently the product of former inflammation.* In the immediate neighbourhood of the dilated tubes, however, it was solid, but of a red colour and soft consistence, the consequence of more recent inflammation; the heart was more than twice its usual size, the right ventricle greatly enlarged and thickened, the left thickened without alteration of its capacity. Dilatation of the auricles. No disease of the valves. Aorta healthy."
85.

This man had been sent into the hospital for hydrothorax, of which he had most of the usual external symptoms. The stethoscope shewed, at once, that this was not the case. The diminished respiratory murmur, combined with the hollow

* "*L'Induration gris*" of Andral, Clinique Medicale tom. 11.

sound on percussion, proved the emphysema, and shewed that no effusion had taken place. We strongly recommend the following observations to the serious attention of our brethren.

" With reference to this and the first case, we may observe, that no practical error is of more frequent occurrence than the attributing to hydrothorax, symptoms which depend upon pneumonia. This error occurs repeatedly in Maclean on Hydrothorax, many of the cases he relates in the Appendix being evidently cases of pneumonia. Thus in case 35, (Appendix,) we find the most unequivocal symptoms of frequently recurring attacks of pneumonia during life, and the post mortem examination exposes hepatization of the lungs of various degrees, and great extent, evidently the consequence of repeated pneumonia; notwithstanding which the case is related as affording an instructive example of hydrothorax! And why? Because, forsooth, the left cavity of the thorax contained more than a pint and a quarter of a yellowish fluid. This is in truth to mistake the consequence for the cause.

" Case XIX. (Appendix p. 33) is likewise a case of pneumonia, treated as simple hydrothorax; we need not therefore wonder at its rapid and fatal termination, 'although the habit was placed under the full influence of digitalis.'

" We wish to call the attention of the profession to this subject, because, with a few exceptions, the great mass of practitioners still adhere to the Cullenian definition of hydrothorax, in apparent ignorance of its overthrow by Corvisart and Laennec.

" In proof of this assertion we may state, that numerous cases have been sent into the medical wards of the Meath Hospital, by practitioners who had named and treated them as cases of simple hydrothorax; but in no instance have we found this diagnosis correct, and more than once have we succeeded in saving the life of such a patient, by the bold use of the lancet, at a period of the disease when a reliance on antidropsical remedies alone would have been of no avail.

" Numerous dissections made during the last five years in the Meath Hospital have convinced us, that although many die *with* hydrothorax, few can be said to die *of* it; at least we have not as yet met with a case of hydrothorax unaccompanied by evident marks of lesions, either in the heart or lungs, *of a date previous to the occurrence of effusion into the cavity of the chest.*

" In the dissection of such cases, the presence of so much fluid in the chest was formerly thought quite sufficient to account for all the symptoms, so that when water was found, the thoracic viscera were examined either very superficially, or not at all; and consequently the real root of the evil was, in most instances, overlooked. The false pathological opinions which sprung from this error, appeared the more plausible, because the supposed gradual accumulation of water in the chest from the very commencement of such a disease, seemed quite adequate to produce that derangement in the function of respiration and circulation, which we know depends on quite another cause, viz. disease of the lungs or heart. In the last edition of Dr. Good's Study of Medicine, Vol. V. p. 404, we find the following definition of *Hydrops Thoracis:*

" 'Sense of oppression in the chest: dyspnœa in exercise or decumbiture: livid countenance: urine red and spare: pulse irregular: œdematous extremities: palpitations and startings during sleep.'

" Now we have no hesitation in affirming, that were we to meet with a case answering to this definition in *every particular*, we would at once declare the disease *not to be* hydrothorax; for in every such case, a careful examination of the chest by means of percussion and the stethoscope, will detect diseases in the thoracic viscera of prior occurrence to the effusion, *when effusion really exists;* and in many cases, when several of the above enumerated symptoms occur, they will be found on examination to commence long before effusion into the chest has taken place.

" On the whole, we feel convinced that the opinions advanced by Corvisart and Laennec, concerning the extremely rare occurrence of idiopathic hydrothorax, are correct; and we cannot therefore subscribe to the reasons assigned by Dr. Good, for still retaining the old definition of that complaint, a definition completely at variance with our dissections." 89.

### Art. VI.

*A Case of Ununited Fracture of the Tibia, treated successfully by the Seton.* By John Browne, M. D. Surgeon to the County Meath Hospital.

This practice has not yet established its reputation upon a firm basis. It is of recent origin, and it has sometimes failed—even where it was said to have succeeded. The measure therefore needs confirmation.

*Case.* Matthew Fitzpatrick, aged 60, a gardener, of a naturally good constitution, received a compound fracture of both bones of the leg, on the 3d of August, 1825, from the passage of a cart-wheel over the limb. The fractures were oblique—situated about the centre of the bones—and half an inch of the spiculated extremity of the upper portion of the broken tibia projected anteriorly through a small wound. He was under a bone-setter for five weeks, during which considerable inflammation occurred and abscesses took place. On the 7th September he was received into hospital—his general health much impaired—pulse 100—no appearance of union in the fracture. There were two small openings communicating with the fracture, and discharging moderately. The foot was œdematous. By proper position, splints, diet, and regimen, the health improved, and by the 5th November the fracture of the fibula had united firmly; but between the ends of the tibia there was a soft union, allowing of motion. Health good. The case was considered favourable for the seton, and the operation was performed, in the following manner.

"A common silver probe was first introduced into the opening on the inside of the tibia, and passed through the course of the fracture; its end being then felt at the outer side of the tibia under the integuments, it was cut upon, and brought out; a curved steel probe, armed with a waxed thread seton, was then introduced, and the silver probe withdrawn. The seton passed through at about one-third of the depth of the bone, it being found impracticable to pass it deeper. The diet and remedies before-mentioned were continued." 322.

The seton was moved thrice weekly from the time of the operation; and we find, on the 17th December, that the patient had, for a week, experienced much irritation, pain, and interruption of rest. The openings were spongy, and the seton was no longer in the channel of the tibia, but confined merely by a portion of integuments. The bone was no longer denuded, and a depression which had existed on its anterior surface since the injury appeared considerably diminished. The limb felt stronger. The seton was removed. By the 19th January, 1826, the tibia was firmly united. Dr. Browne observes that—"the rapidity with which the deposition of bone occurred in this old man was surprizing, and it was extremely interesting to see the seton gradually pushed forward by the efforts of Nature, in proportion as the cavity in the bone became filled up."

We must defer the conclusion of the review of this volume till our next number, when we hope to complete it.

